# Pancreatic Cancer Signaling Pathways, Genetic Alterations, and Tumor Microenvironment: The Barriers Affecting the Method of Treatment

**DOI:** 10.3390/biomedicines9040373

**Published:** 2021-04-02

**Authors:** Darya Javadrashid, Amir Baghbanzadeh, Afshin Derakhshani, Patrizia Leone, Nicola Silvestris, Vito Racanelli, Antonio Giovanni Solimando, Behzad Baradaran

**Affiliations:** 1 Immunology Research Center, Tabriz University of Medical Sciences, Tabriz 5166614766, Iran; darya.r767@gmail.com (D.J.); amirbaghbanzadeh@gmail.com (A.B.); afshin.derakhshani94@gmail.com (A.D.); 2 IRCCS Istituto Tumori “Giovanni Paolo II” of Bari, 70124 Bari, Italy; n.silvestris@oncologico.bari.it; 3 Guido Baccelli Unit of Internal Medicine, Department of Biomedical Sciences and Human Oncology, School of Medicine, Aldo Moro University of Bari, 70124 Bari, Italy; patrizia.leone@uniba.it (P.L.); vito.racanelli1@uniba.it (V.R.); 4 Department of Biomedical Sciences and Human Oncology, School of Medicine, Aldo Moro University of Bari, 70124 Bari, Italy; 5 Department of Immunology, Faculty of Medicine, Tabriz University of Medical Sciences, Tabriz 5166614766, Iran

**Keywords:** pancreatic cancer, pancreatic adenocarcinoma, PDAC, immune checkpoint, signaling pathway, gene mutation

## Abstract

Genetic alterations, especially the K-Ras mutation, carry the heaviest burden in the progression of pancreatic precursor lesions into pancreatic ductal adenocarcinoma (PDAC). The tumor microenvironment is one of the challenges that hinder the therapeutic approaches from functioning sufficiently and leads to the immune evasion of pancreatic malignant cells. Mastering the mechanisms of these two hallmarks of PDAC can help us in dealing with the obstacles in the way of treatment. In this review, we have analyzed the signaling pathways involved in PDAC development and the immune system’s role in pancreatic cancer and immune checkpoint inhibition as next-generation therapeutic strategy. The direct targeting of the involved signaling molecules and the immune checkpoint molecules, along with a combination with conventional therapies, have reached the most promising results in pancreatic cancer treatment.

## 1. Introduction

Because of its poor prognosis, pancreatic cancer causes almost as many deaths (466,000) as cases (496,000) and is the seventh leading cause of cancer death in both men and women. Higher human development index (HDI) countries have rates that are four to five times higher, with the highest incidence rates in Europe, Northern America, and Australia/New Zealand. In a survey of 28 European countries, it was expected that pancreatic cancer would overtake breast cancer as the third leading cause of cancer death by 2025, owing to its relatively stable rates compared to the decreasing rates of breast cancer. [1] The persistent increase in the prevalence of pancreatic ductal adenocarcinoma (PDAC) will turn it into the second top cause of cancer-associated losses in the US in the near future [2]. One of the origins that underlies this lethal phenotype is a vital mechanism that may comprise the takeover of innate cellular functions to maintain PDAC growth. For instance, quite a few researchers have discovered that chemokine receptors expressed on PDAC cells heighten invasiveness and growth, which is in striking divergence to their instinctive functions through organogenesis and inflammatory response [3,4,5,6]. Non-biomarker-driven combination chemotherapy is of marginal benefit in pancreatic cancer [7,8]. Nonetheless, selected patients’ subsets can be potentially chosen and maximally benefit from a given therapy, converting a one-size-fits-all approach to an individualized biomarker approach to cancer therapy. The first example of a biomarker identified subpopulation of pancreatic cancer patients that can drive therapeutic decision making is represented by BRCA1, BRCA2, or PALB2 mutations. In this context, platinum-based therapy with or without a PARP inhibitor has achieved a significant response [9,10]. Therefore, it is vital to thoroughly detect the population of patients who have a BRCA-like phenotype in this disease. However, the challenges in pancreatic cancer regarding molecular phenotyping are extreme. The specimens are typically small fine-needle aspiration biopsy materials. PDAC is a tumor with a markedly expanded stroma and is characterized by typically very low cellularity.

Additionally, the complexity of providing timely molecular phenotyping in a clinically relevant period is remarkable. The pivotal task of the immune system in the control and extermination of different cancers is undeniable [11]. Whole-genome sequencing of pancreatic cancers revealed that 119 somatic chromosomal structural variants were found in each patient. This is an overstatement, since researchers believed that the number of mutations already exceeds 63 [12]. Most of the structural variants include intra-chromosomal deletions, tandem duplications, inversions, amplified inversions, chromosomal rearrangements, and fold back inversions, and are involved in 12 diverse core-signaling pathways at a minimum, which was restructured in 67% to 100% of malignancies [13]. Famous genetic mutations, including KRAS, TP53, CDKN2A, and SMAD4,2, are noteworthy in PDAC (with KRAS including more than 90% of the mutations). Moreover, T cell immunity in human cancers can recognize tumors by identifying tumor-specific neoantigens [14]. In the case of PDAC patients, it is considered that K-Ras mutations not only are the reason for the initiation of cancer but also immediately trail other mutations, which contributes to the aggressive nature of pancreatic tumors [15].

In the environment of the tumor, the various mechanisms of immune suppression may occur to avert effective antitumor immunity [11]. In order to survive through the cytotoxic T-cell activity, PDAC, alongside several other cancers, also takes up the inhibitory effects of immune checkpoints [16]. Immune checkpoint inhibitors, targeting immune checkpoints like PD-1/PD-L1 and CTLA-4, have shown to be noteworthy pathways impending in quite a few forms of malignancies together with PDAC. It is supposed that tumor cells evade immune responses via dodging checkpoint control, which in blocking the inhibitor activity of T-cell mediated immune response further improves the immune system’s responses to fight the tumor [17]. This review will focus on different types of mutations, signaling pathways, along with a glance at immune system-based therapies, as well as potential therapeutic targets in pancreatic ductal adenocarcinoma.

## 2. Signaling Pathways

### 2.1. K-Ras Oncogene

K-Ras point mutations are present in most PDAC patients. They are the most primitive genetic alterations that originated in early pancreatic lesions such as low-grade PanIN [15]. To have a constant proliferation and survival, pancreatic cancer cells need continuous K-Ras signaling [18]. Ras proteins are members of a small G protein superfamily, and guanine nucleotides like GTP and GDP regulate their activity. As a result of Ras binding to GTP, Ras downstream signaling pathways are triggered. GEFs and GAPs regulate the Ras signaling active and inactive states. In the process of GDP/GTP interchange, GEFs act as a catalyzor which exchanges bound GDP with cytosolic GTP. GAPs activate the intrinsic GTPase activity of Ras protein to hydrolyze GTP into GDP [19]. Any mutations that inactivate the GTPase constitutively activate Ras signaling and downstream effector pathways. In order to grow acinar to ductal metaplasia and PanIN, the mutations in codons G12D or G12V are sufficient to bring about PDAC progression. Further mutations on tumor suppressors such as P16/CDKN2A, SMAD4, and p53 as well as the progression of cancers arising from a positive K-Ras mutation can be improved in mice. The identified tumor suppressor gene mutations repeatedly occur in precancerous pancreatic lesions [19].

Numerous downstream effectors are engaged through K-Ras signaling. In pancreatic cancer, it predominantly acts through canonical Raf/MAPK/extracellular signal-regulated kinase (Erk), PI3Ks/(PDK-1)/Akt, RalGEFs, and phospholipase Cε [19]. In the case of alterations or mutations in the mentioned downstream pathways, K-RAS-driven PDAC gets complicated. For instance, the expression of a constitutively active oncogenic class 1A PI3K (PI3CA H1047R) in Ptf1a positive cells leads to the induction of acinar to ductal metaplasia, premalignant PanIN, exclusion of PDK-1, and blocking of K-RasG12D driven PDAC [20].

### 2.2. Tumor Suppressor Genes: TP53, SMAD4/DPC4, and P16/CDKN2A

In pancreatic cancer, the most frequently active tumor suppressor gene is P16/CDKN2A, which blocks CDK4/6 mediated phosphorylation of retinoblastoma, which blocks entry into the S phase of the cell cycle. Different mechanisms lead to the inactivation of P16/CDKN2A, including epigenetic silencing by promoter methylation, homozygous deletions, and loss of its heterozygosity [21]. As for K-Ras and P16/CKN2A, they cooperate in the development of PDAC. Selective pressure is exerted as an effect of the mutations in K-Ras, resulting in the subsequent mutation in P16/CDKN2A, which gives birth to PDAC development. P16/CDKN2A and oncogenic K-Ras expression co-exist alongside other aging markers, whereas in pancreatic cancer, the expression of P16/CDKN2A and markers are omitted [22].

In 50% to 75% of PDAC patients, P53 is inactivated, and this happens through the combined loss of second allele and intragenic mutations. P53 mutations result in the loss of function and, consequently, survival and the growth advantage for the cells with chromosomal aberrations in the late PanIN stage available [23].

SMAD-4 is a key signal transducer of the TGF-β signaling pathway, and it is inactivated in nearly 55% of pancreatic cancer patients. The inactivation occurs either via homozygous deletions or by intergenic mutations and the second allele’s loss. As a result of SMAD4 loss, the advantage of growth for pancreatic cancer cells in the late PanIN stage (PanIN-3) is provided by nullifying the signals arbitrated by TGF-β [24]. In SMAD4 expression, the PDAC patients who had undergone surgical resection survived for longer, as noted by some studies [25].

### 2.3. Signaling of Growth Factor Receptors

There are several mitogenic growth factors and their ligands, which are overexpressed in pancreatic cancer, namely: EGF and EGFR (receptor of EGF), multiple EGFR binding ligands; FGF and FGFR; IGF and its receptor (IGFR); PDGF; VEGF [26].

#### 2.3.1. EFGR

EGF receptor (EGFR) is an intracellular receptor-activated through binding its ligands EGF and TGF-α. In 90% of pancreatic tumors, EFGR overexpression was identified and played a significant part in the recurrence of human pancreatic cancer and liver metastasis. The inhibitors of EGFR inhibited orthotopic tumors’ growth combined with chemotherapy and decreased tumorigenesis and PDAC cell growth in vitro. Conversely, a combination of EGFR targeting agents did not offer many clinical benefits in pancreatic cancer patients [27,28].

#### 2.3.2. IGF

In various cancers, the insulin-like growth factors and their receptors have been identified as pivotal factors by regulating angiogenesis, invasion, and cell survival [29]. Prominent expression of IG-F1 and IGF1R is linked with poor survival and a higher tumor grade in PDAC patients [30]. Exogenously added IGF-1 heightened the pancreatic cancer cells’ growth in vitro, and its influence was repressed by the antibody that neutralizes IGF-1. Nonetheless, IGF1R blocking antibodies were unsuccessful in clinical trials. Amgen announced that a great phase III clinical trial in metastatic pancreatic cancer cases treated with the ganitumab (an antibody that blocks IGF1R) plus gemcitabine had been interrupted because the above assortment was not successful in increasing overall survival in comparison to gemcitabine alone [31].

#### 2.3.3. FGFR

Upon binding to FGF, FGFR triggers phosphorylation of FGFR substrate 2. PI3K/Akt and Ras/MAPK pathways elements are recruited and activated via phosphorylated FRS2. In a subclass of pancreatic cancers, overexpression of FGFR-1 and FGFR-2 receptors and their ligands (FGF1-7) contribute to angiogenesis and mitogenesis. In preclinical pancreatic cancer models, a tyrosine-kinase inhibitor, shRNA, or dovitinib was used to inhibit the FGFR signaling, and it achieved remarkable anti-cancer effects [32].

#### 2.3.4. VEGF

VEGF stimulates endothelial cell proliferation and survival via binding to VEGFR-1 and VEGFR-2 (its receptors). An elevated expression of VEGF mRNA was observed in PDAC patient tumor samples. It was correlated with disease progression and great microvessel density, although PDAC is not an extremely vascularized tumor [33,34]. In the subcutaneous mouse model, TNP-40, which is an analog of an anti-angiogenic agent (fumagillin), decreases tumor growth and metastasis of the cell lines of PDAC [35]. In preclinical pancreatic cancer models, the adenoviral vector that carries the PTK 787 (inhibitor of VEGFR tyrosine kinase) similarly hindered the metastasis and growth of PDAC [36].

#### 2.3.5. RAGE

The receptor for advanced glycation end products (RAGE, also known as AGER) is a transmembrane immunoglobulin superfamily receptor encoded in the major histocompatibility complex’s Class III region. RAGE activation promotes inflammation and has been linked to a variety of chronic diseases, including diabetes, neurodegenerative disorders, and cancer. RAGE plays a special role in pancreatic tumorigenesis and drug resistance, as was recently discovered. In vitro and in vivo, knocking down or knocking out RAGE delays the growth of oncogenic KRAS-driven pancreatic tumors and reverses drug resistance. In pancreatic ductal adenocarcinoma tumor cells, previous findings demonstrated that RAGE modulates crosstalk between pro-survival pathways, IL6-pSTAT3, and autophagy, and contributes to the development of early pancreatic intraepithelial neoplasia. RAGE is needed for oncogenic KRAS-mediated hypoxic signaling in pancreatic cancer, according to Kang et al. In another study, they also revealed that by regulating mitochondrial bioenergetics, the HMGB1/RAGE inflammatory pathway promotes pancreatic tumor development. As a result, targeting RAGE for pancreatic cancer treatment is a novel approach [37].

### 2.4. EMT in PDAC

**Figure 1:**
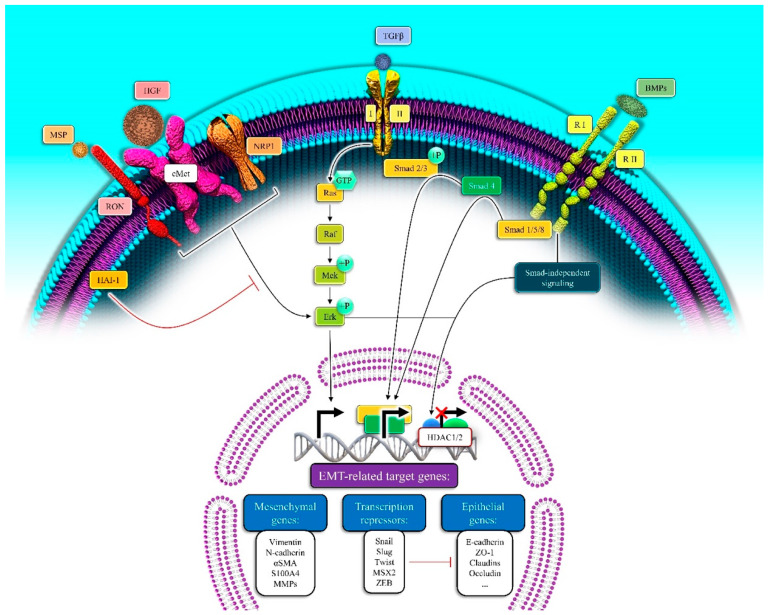
The induction of EMT by targeting EMT-related genes via activation of the growth factor signaling pathway in pancreatic cancer. The binding of the growth factors to their related receptors triggers the EMT-related genes’ expression. MSP: Major Surface Protease, IGF1: Insulin-like growth factor 1, TGFβ: Tumor growth factor β, BMPs: Bone Morpho-genetic Proteins, RON: Recepteur d’Origine Nantais, NRP1: Neuropilin1, Ra1: Retinoic Acid Induced 1, Mek: The mito-gen-activated protein kinase kinases (the MAPK/ERK kinases; MKKs or MEKs), Erk: extracellular signal-regulated kinase, HAI-1: Hepatocyte growth factor activator inhibitor-1, HDAC1/2: Histone Deacetylase 1 and 2, MSX2: Msh Homeobox 2, S100A4: S100 Calcium Binding Protein A4, ZO-1: Zonula occludens-1.

As the cancer process becomes clearer, it seems that the dissemination of pancreatic cancer cells from the primitive tumor and liver metastasis occurs even before the disease is identified, namely at the original state of the disease. Most of the deaths related to pancreatic cancer take place because, in metastatic disease, the EMT carries an essential burden in the rapid tumor progression [38]. Epithelial cells miss their markers like E-cadherin, occluding, claudin, and laminin 1 (epithelial markers) and gain N-cadherin, vimentin, and fibronectin (mesenchymal markers) during EMT progression [39]. There are three key sorts of EMT based on the setting in which it happens. In carcinoma cells, type 3 EMT exists and is considered necessary for invasion, intravasation, extravasation, spread, and metastasis during the tumor growth process [40]. EMT in tumor progression is directed by a multipart network of signaling pathways. EMT during cancer progression is guided by both non-soluble components (hyaluronic acid and collagen) and soluble factors of the extracellular matrix (Wnt, FGF, HGF, Notch, TGF-β family members, TNF-α and HIF1-α). The induction of transcription factors such as Snail 1 and 2, Zeb-1 and 2, and family members of bHLH (E12, E-47, and Twist) are the primary signaling events that induce EMT. The repression of the encoder of E-cadherin (CDH1 gene) is the common feature of the pre-mentioned transcription factors. Therefore, it is an important primary step in the trans-differentiation of epithelial to a mesenchymal phenotype, invasion, and metastasis [41], as seen in Figure 1.

#### 2.4.1. TGF-β Signaling Pathway in EMT

In several tumors, including pancreatic cancer cells, TGF-β is one of the major EMT-inducing factors. When TGF-β binds to a type II receptor, it allows the trans-activation of TβR I (type I receptor) in canonical TGF-β signaling. TβR I (a serine/threonine kinase) phosphorylates SMAD2, and the subsequent 3 form a complex with SMAD4, with translocation to the nucleus regulating the transcription of the target genes. As for TGF-β mediated EMT induction, the transcriptional activation of Snail, Zeb-1, Slug, and Twist, seems important [42].

TGF-β may function via a non-canonical pathway such as a SMAD-independent pathway, which involves PI3K, ERK/MAPK, p38, RhoA, JNK, and additional pathways of signaling in PDAC [43]. SMAD4 depletion in the Colo-357 pancreatic cancer cell line with RNAi did not succeed in disturbing EMT responses in those cell lines [44]. TGF-β-induced EMT was inverted by the MEK-1 inhibitor PD98059 in other pancreatic cancer cell lines [45].

#### 2.4.2. Wnt/β-Catenin Signaling Pathway

In the canonical Wnt/β-catenin signaling pathway, β-catenin is held down via a demolition complex including Axin, Adenomatous Polyposis coli, glycogen synthase kinase-3β, and CK-1, in the lack of Wnt ligand. First, it is the CK-1 phosphorylates β-catenin at Ser45, that begins its initial response. GSK-3β phosphorylated primed β-catenin at Thr41, Ser33, and Ser37, eventually leading to ubiquitination and proteasomal degradation by β-Trcp. β-catenin nuclear accumulation is prohibited by the persistent removal of β-catenin, where Wnt target genes are repressed by DNA bound TCF/LEF and HDAC. The complex which results in phosphorylation of LRP5/6 is formed upon Wnt ligand binding to Frizzled and LRP5/6 receptors. Phospho LRP5/6 binds the axin, thus stabilizing and facilitating the dismantling of the GSK-3β complex and inactivating cytosolic β-catenin. β-catenin can form a complex with TCF/LFE, and therefore controlling the activation of genes required for cell growth and proliferation in the nucleus [46].

GSK-3β promotes snail phosphorylation and proteasomal degradation. Their activity is suppressed by Wnt, which also alleviates the protein levels of Snail. Wnt induces stemness and EMT in cancer cells. K-Ras activates the Wnt/β-catenin signaling pathway, which up-regulates the stimulators of EMT [47]. Wnt/β-catenin signaling pathway inhibition prevents epithelial-to-mesenchymal transition. Restoration of Wnt inhibitory factor 1 leads to reduced expression of mesenchymal markers and increases expression of epithelial markers through Slug and Twist reduced expression. Knockdown of β-catenin via small hairpin RNA leads to overexpression of E-cadherin. It reduces expression of the mesenchymal markers vimentin, N-cadherin, and MMP-2, indicating the reversal of epithelial-to-mesenchymal transformation [48].

#### 2.4.3. Signaling Pathway of Notch

The signaling through the Notch pathway plays an important role in differentiating the tissue and also the cell death [49]. As of yet, four Notch receptors and five Notch ligands (Delta-like 1, 3, 4, and Jagged-1 and 2) have been identified by researchers. Activation of Notch signaling happens when the Notch protein binds to a receptor on the adjacent cell. The enzyme Notch aims to be cleaved over a cascade of proteolytic cleavages by the metalloproteases, tumor necrosis factor alpha-converting enzymes, and γ-secretase. Gamma secretase complex releases active Notch intracellular domain fragment (NICD). This fragment (NICD) is translocated into the nucleus and then binds to the transcription factor CSL (CBF1, Suppressor of Hairless, and Lag-1). The complex of CSL-NICD acts as a co-activator recruiting another co-activator complex encompassing p300 and other co-activators, resulting in the activation of Notch target genes that are essential in the regulation of cell growth, proliferation, angiogenesis and apoptosis (e.g., Cyclin D1, COX-2, Akt, MMP9, ERK, VEGF, c-Myc, mTOR, NF- kB, p53, p27, and p21) as in other solid and hematological malignancies [50,51,52]. The Notch pathway directly up-regulates Slug and Snail-1, triggering the epithelial to the mesenchymal transition. Notch-2 or midkine (a downstream target of Notch-2) knockdown induces EMT inhibition in pancreatic cancer cells [53].

#### 2.4.4. Snail Transcription Factors

Snail (Snail-1), Slug (Snail-2), and Smuc (Snail-3) are the Snail transcription factors. Slug and Snail activate the EMT during developing and pathological conditions. Having exceedingly conserved C2H2 type, zinc finger motifs seem to be the most common characteristic of all these transcription factors. In order to maintain transcriptional repression of target genes and protein stability, the Snail1/GFI domain at the amino terminus is essential [54]. In pancreatic tumor progression, Snail and Slug are the primary mediators of EMT. In 50 percent of cases, Slug was expressed, and modest to robust Snail expression was in 68% of PDAC patients. In pancreatic cancers, high levels of Snail expression associated with lymph node invasion and metastasis to distant areas [55]. In an orthotopic mouse model of pancreatic cancer, Snail-transfected cell lines of pancreatic cancer showed highly invasive and metastatic capacities. Cell lines of pancreatic cancer were enabled to go through EMT at the tumor’s invasive stage [56]. Knocking out Snail enhanced sensitivity to gemcitabine, leading to the amplified overall survival in a genetically modified mouse model of pancreatic ductal adenocarcinoma [57]. Snail represses the genes involved in maintaining epithelial phenotype (occludin, E-cadherin, claudin, and cytokeratin-18). Expressions of mesenchymal genes such as N-cadherin, vimentin, and fibronectin, for example, are triggered by Snail. Moreover, Snail has been shown as the regulator of genes involved in apoptosis (P53, BID, and DFF40) and cell polarity (Crumbs3, Lgl2, and dlg3), among them the only direct target of Snail is E-cadherin [58].

#### 2.4.5. Zeb Transcription Factors

The Zeb family of transcription factors is one of the best evaluated EMT induction agents. Both tumor-associated stroma and pancreatic cancer cells demonstrated a great level of Zeb-1 expression, which was linked with poor prognosis in PDAC patients. In human tissue samples and pancreatic cancer cell lines, there was an inverse relationship between Zeb-1 and E-cadherin expression [59,60]. Cell migration, tumorigenesis, and dissemination have been reduced after silencing Zeb-1 [61]. It has been shown that Zeb-1 reduces the expression of main components of epithelial differentiation, cell adhesion, and cell polarity, which is a well-studied consequence of Zeb-1 expression. This TF declines E-cadherin expression by recruiting HDAC-1/2 or Switch/sucrose non-fermentable chromatin remodeling protein BRG1 to the promoter region of the CDH-1 gene [60]. Therefore, the Zeb-1 inhibitor drugs might have clinical significance for PDAC patients.

#### 2.4.6. bHLH Transcription Factors

Between the studied bHLH transcription factors, E12, E47, Twist 1, and Twist 2 play pivotal roles in EMT [62]. E-47 and E-12 are repressors of E-cadherin expression and prompt EMT [63]. The primary regulators of EMT during pathogenesis are Twist 1 and Twist 2 [64]. In tissue samples from PDAC patients, the expression of Twist is either absent or very weak. Low expression of Twist has also been observed in PANC-1, MiaPaCa-2, Capan-1, AsPC-1, and HPAF-2 cell lines of pancreatic cancer; still, induction of Twist expression occurs under hypoxic conditions, and this, seemingly, may take part in the invasive nature of tumors of pancreas [65]. Twist reduces E-cadherin expression but induces the expression of N-cadherin [66]. It has been shown that the interaction of Twist using a number of constituents of Mi2/nucleosome remodeling and deacetylase complex represses the E-cadherin transcription [67].

## 3. The Tumor Microenvironment (TME)

A TME is a sophisticated arrangement with atypical physical and biochemical characteristics, wherein collaborations amongst cancerous and stromal cells encourage disease progression, carcinogenesis, therapeutic resistance, and metastasis [68,69]. Other than the resistance deliberated by desmoplasia, pancreatic cancer is considered to have an exceedingly immunosuppressive environment, with numerous constituents and pathways hindering influential pancreatic cancer-targeted immune responses [70]. Recognition of the principal TME mechanisms is puzzling because of the heterogeneous nature of PC stroma that is infiltrated with various immune regulatory cells. The constituents of the tumor microenvironment (TME) are immune cells, pancreatic stellate cells, acellular stroma, and soluble factors. PDAC is marked by a stromal reaction known as desmoplasia, in which overactive cancer-associated fibroblasts deposit an excessive amount of extracellular matrix (ECM), the majority of which is fibrillar type I collagen. This stromal remodeling and dysregulation of cell-ECM homeostasis is thought to lead to cancer progression, including metastasis and drug resistance. Clearly, tumor-stromal ECM interactions are important in PDAC pathophysiology; however, more advanced in-vitro and in-vivo models are needed to gain a better mechanistic understanding of the disease. Basement membrane (BM) and interstitial matrix (IM) are two forms of ECM that are associated with PDAC as well as normal tissues. BM is a thin sheet-like structure composed mainly of laminin, non-fibrillar type IV collagen, and heparan sulfate proteoglycan that protects and polarizes epithelial cell layers while separating them from the underlying interstitial tissue compartment. Specific mesenchymal cells (e.g., fibroblasts) reside within fibrillar type I collagen, which is the most prominent component of IM [71]. In a study, the authors discovered collagen density as a novel regulator of anti-cancer T cell activity using 3D T cell culture. This immunosuppressive mechanism may be critical for cancer cells evading immune destruction, and it may be a new therapeutic goal for improving immunotherapy efficacy. Also, they revealed that collagen density regulates the activity of tumor-infiltrating T cells. A high-density matrix induces downregulation of cytotoxic activity markers and upregulation of regulatory T cell markers, according to whole-transcriptome analysis of 3D-cultured T cells. Their research uncovers a novel immune modulatory mechanism that could be important for T cell suppression in the tumor microenvironment [72].

Fibronectin has some similarities to collagens, it also has its own unique effect on PDAC biology. Moreover, fibronectin contains collagen-binding sites, making it a linker protein between collagens and integrins that supports collagen function. PDAC cells infiltrate the basement membrane after being irradiated, which can be prevented by using integrin 5-1 blocking antibodies or fibronectin depletion. Surprisingly, fibronectin appears to play a key role in PSC ECM synthesis. Fibronectin binds to the latent TGF binding protein, allowing active TGF to be released, which activates PSCs. As a result, fibronectin is an important component of the ECM, facilitating both PDAC cell malignancy and fibrogenesis [73].

Hyaluronan (HA), one of the main ECM components found in tumor stroma, has been extensively studied in relation to cancer progression. The amount of HA generated in the body is regulated by a balance of synthesis and degradation in normal physiological conditions; however, HA has been found to be abundantly accumulated in the stroma of malignant tumors. The presence of HA in the microenvironment can help tumor progression by promoting cell proliferation, migration, invasion, metastasis, angiogenesis, and chemotherapeutic resistance. HA and its receptors have been shown to be overexpressed in PDAC in many studies. Importantly, irregular HA accumulation is related to a worsening prognosis in PDAC patients. The accumulation of extracellular HA caused by forced expression of synthesizing genes stimulated tumor growth in an experimental model of PDAC. These results indicate that HA may play a key role in the development of PDAC and may be a therapeutic target [74].

The continuous cell-cell and cell-matrix interactions maintain the TME. In the progression of PC, the induction of TME is important, because it leads to drug resistance. The induction of TME happens during the interaction among epithelial cells, pancreatic cancer cells, and stromal cells. TME components can also result in the formation of connective tissue in primary and metastatic sites or promotion of the metastatic ability of PC by augmenting EMT and angiogenesis [75].

Moreover, TME is a hurdle in the immunotherapeutic strategies. Tumor-infiltrating lymphocytes (TILs) are genetically divergent immune cells interlinked with TME, wherein CD8+T lymphocytes and CD4+ helper T1 lymphocytes are interlinked with satisfactory outcomes. In contrast, CD4+ helper T2 lymphocytes have adverse effects on patient survival [76]. Immune and Inflammatory cells are fundamental elements in the pancreatic cancer TME and create chemo-resistance turned into an intense research field. Early carcinogenesis and metastasis occur as a result of the abundance of inflammatory cells in the pancreatic stroma. These cells play roles in both fibrosis and neovascularization [77].

## 4. Immune Checkpoints

Immune checkpoint proteins are surface molecules on immune effector cell masses that activate or inhibit effector function when linked to their associated ligands. Expression of the co-inhibitory ligands on cancer cells has been recommended as a mechanism by which these cells dodge the immune response. IRs (inhibitory receptors) are mediators of T cell dysfunction in autoimmunity and chronic disease and play a substantial role in modulating immune response [78,79]. Targeting molecules, such as CTLA-4 and PD1 in cancer patients, can revitalize the antitumor immune response, as has already been shown in some clinical trials [80,81,82].

### 4.1. CTLA-4

CTLA-4 is a homolog of CD28 with a higher affinity of binding to B77,8. Unlike CD28, the competitive binding of B7 and CTLA-4 does not result in the production of a stimulatory signal, and it can avert the costimulatory signal generally delivered by the binding of CD28:B7 [83,84]. The comparative binding extent of CD28:B7 as against CTLA-4:B7 is a determiner for a T cell to endure anergy or activation [85]. Direct inhibition at the T cell receptor synapse, inhibition of CD28 or its signaling pathway, and augmented mobility of T cells resulting in a reduced capacity to cooperate with APCs are the anticipated mechanisms for inhibitory signals [86,87].

Predominantly via localization within the cell, CTLA-4 is subject to regulation and is located mainly in the intracellular compartment in resting naïve T cells. Stimulatory signals induce upregulation of CTLA-4 on the surface of the cells from both TCR and binding of CD28:B7 via exocytosis of CTLA-4 encompassing vesicles. A feedback loop in which resilient TCR signaling stimulates more translocation of CTLA-4 to the cell surface operates the mentioned process [88].

CTLA-4 also plays its part in extra aspects of immune control. In contrast to effector T cells, Tregs constructively express CTLA-4 and control functions of the effector T cells. Tregs are the main performers in retaining peripheral tolerance, and the CTLA-4 expression seems to be important for the regulatory T cells’ suppressive functions. Genetically CTLA-4 deficient Tregs diminished their suppressive functions in animal models. Downregulation of B7 ligands on APCs, resulting in decreased CD28 co-stimulation, is a probable mechanism whereby Tregs control effector T-cells [89].

### 4.2. PD-1

PD-1 is another member of B7/CD28 costimulatory receptors, and it regulates T cell activation via binding to PD-L1,2. PD-1 binding inhibits IFN-γ, IL-2, and TNF-α production. Like CTLA-4 signaling, it also inhibits T-cell proliferation and reduces survival of T-cell [90]. It is assumed that the expression of PD-1 is a hallmark of exhausted T cells as a result of high stimulation or reduced CD4+ T-cell help. Characterized by T-cell dysfunction, a state of exhaustion occurs during cancer and chronic infections, leading to suboptimal control of such diseases [91].

Both PD-1 and CTLA-4 have parallel adverse consequences on the activity of T cells. The responsible signaling mechanisms, the timing of downregulation, and the anatomic locations of immune inhibition via the two immune checkpoints are different. PD-1 is mostly expressed on B cells, activated T cells, and myeloid cells [92], and it functions during the effector phase, principally within peripheral tissues [90]. PD-L1 and PD-L2 are more extensively expressed compared to the CTLA-4 ligands [93]. The PD-1-PD-L1/PD-L2 interactions are considered to preserve tolerance inside locally infiltrated tissues due to the fact that PD-1 ligands are expressed in peripheral tissues [92]. PD-1 is expressed on Tregs, but the function of this expression on these cells remains imprecise. The binding of PD-1 to its ligands reduces the immune response in T-cells previously engaging in an effector T-cell response [94].

### 4.3. PD-L1

One of the essential ligands of PD-1 is PD-L1, and it is expressed on nonhematopoietic cells, nonlymphoid tissues, and leukocytes. PD-L1 can be induced on parenchymal cells via tumorigenic signaling pathways or inflammatory cytokines such as IFN-γ [95]. The expression of PD-L1 has been detected in numerous and diverse kinds of cancers and is correlated with an increased number of TILs and poor prognosis [96,97,98]. Inhibition of T-cell responses and the conversion of naïve CD4+ T-cells to Treg cells are the contributions of PD-L1, which promote the induction and maintenance of Tregs [99,100]. A study has demonstrated the converse roles of PD-L1 and PD-L2 signaling in the activation of NKT cells [101]. PD-L1 binding to CD80 inhibited T-cell responses, whereas inhibition of PD-L2 binding has resulted in heightened TH2 activity [102,103].

### 4.4. LAG3

Clinically, LAG3 AKA CD223 is one of the most promising new inhibitory receptor targets. It is an immune checkpoint receptor expressed by exhausted and activated CD4+ and CD8+ T cells and Tregs [104]. LAG3 operates functionally by conveying inhibitory signals that regulate immune cell homeostasis, T-cell activation, proliferation, cytokine production, cytotoxic activity, and other functions [79]. Rapid, immune-mediated tissue damage is present in the setting of chronic autoimmunity where LAG regulated homeostasis is disturbed [105,106]. Moreover, insistent stimulation of antigen, like chronic viral infection and cancer, leads to increased levels of chronic LAG3 expression, which results in T cell exhaustion and consequent diminishing of T cell function. Many LAG3 targeting immunotherapies clinically combined with antibodies against other IRs, such as PD-1/PD-L1, are used in cancer treatment [107]. The expression of LAG3 is regulated by activation via the exception of plasmacytoid dendritic cells and Tregs in most cell types, and it exists on chromosome 12 in humans, coding a 498-amino acid protein [104,108,109].

### 4.5. VISTA

VISTA is a negative regulator of T cell, which is expressed on hematopoietic cells. VISTA is a type I transmembrane protein that contains a single N-terminal Ig V-domain, nearly 30 amino acids, a transmembrane domain, and a 95 amino acid cytoplasmic tail [110]. Inside the TME, VISTA levels are increased, and its blockade can improve the antitumor response of the immune system in mice [111]. It has been shown that the IgV domain of VISTA has the maximum homology with PD-L1 but uniquely has four additional invariant cysteines [112]. The conserved cytoplasmic tail of VISTA is similar to CD28 and CTLA-4, but its distinguishing point from other B7 co-receptor molecules is that it does not own a classic ITIM/ITAM motif. Due to some studies, it can be deduced that VISTA can act as both a ligand and a receptor in immune response regulations [110,113,114].

VISTA is principally expressed and up-regulated in the high density-infiltrating immune cells but insignificant in human PC (pancreatic cancer) cells, according to some studies conducted on VISTA expression in pancreatic cancer tissue. Additionally, the VISTA potential as a significant target for PDAC immunotherapy has been evaluated. Blando et al. stated that inhibitory checkpoint inhibition and differential immune infiltration in PDAC compared to melanoma showed that VISTA is a promising target in immunotherapy for PDAC patients [111,115]. Supplementary research is needed to uncover the immunoregulatory mechanism of VISTA in PDAC [116].

## 5. T-Cell Mediated Recognition of Somatic Mutations

Mutational neoepitopes occur through coding mutations and the expression of proteins, which are then cleaved by the immunoproteasome and presented by class I and class II MHC molecules to associated T cells. Self-antigens can also serve as T cell targets in this disease, either chore embryonic antigens that aren’t normally expressed in tumor tissues to tissue-specific antigens, as seen in melanoma, or as a number of other overexpression antigens [117]. Notably, most of the interest in identifying T cell epitopes in cancer has been generated in relation to the potential for somatic mutations to provide effective T cell neoepitopes in this disease.

The recent revolution in immunotherapy has been driven by identifying immune checkpoints, whereby either tumor cells or antigen-presenting dendritic cells can present ligands such as PD-L1 or B7 to checkpoint receptors on T cells, the PD1 receptor, or CTLA-4. Targeting those two receptors has proven to be transformative in treating many patients with solid tumors [14]. While considering the response rates to single-agent immune checkpoint inhibitors, remarkable degrees of response have been detected in melanoma, non-small cell lung cancer, renal cell cancer, and other tumors, with responses to combination immune checkpoint therapy reaching as high as a 60% level of positive outcomes. For gastrointestinal, the responses to combination immune checkpoint therapy were as high as 60%. The response rates have typically been lower for gastrointestinal malignancies in unselected patients in a 20% range (Table 1) [118].

However, in a biomarker-driven manner, the significant advance has recently identified subsets of patients with mismatch repair deficiencies with microsatellite instability. These subjects can achieve remarkably high response rates in colorectal cancer and pancreatic cancer, where the response rate to immune checkpoint risk inhibitors is typically unsatisfactory, except for the very small subset of patients with pancreatic cancer who have a mismatch repair deficiency [119].

Unfortunately, as with metastatic colon cancer patients, as many as 10% may have an unstable microsatellite phenotype. A variable percentage between 1% to 2% is considered to be included in this particular phenotype in pancreatic cancer. Nevertheless, those patients appear to be responsive to immune checkpoint therapies, which can be neglected by treating most patients in a non-biomarker selected way. As the only exception of the microsatellite status, a biomarker has emerged as a potential way to select subsets of patients [120]. Recent efforts tried to uncover alternative biomarkers that might drive the rational application of immune checkpoint therapies, including immunohistochemical determination of PD-L1 expression. This attempt has proved to be imperfect due to the vagaries of different antibodies utilized. PD-L1 can be expressed both on tumor cells, and associated dendritic cells do not help with understanding which is the most appropriate cell type to characterize PD-L1 expression [121]. Other hypermutated phenotypes and PDAC bear mutations in certain DNA polymerases that lead to a high mutational burden, such as POLE mutations [122,123,124].

Additionally, there have been attempts to apply various forms of immunophenotyping, including the well-defined immunoscore, where patients with a higher intrinsic immune response may have higher response rates to immune checkpoint therapies [125]. Furthermore, many recent works demonstrated that the microbiome is an essential determinant of response to immune checkpoint therapies. In experimental models, fecal transplants of certain bacterial species sensitize mice to immune checkpoint therapy [126,127]. Determining the mutational burden through whole-exome sequencing would be an effective determinant of sensitivity to various forms of immunotherapy. However, it very likely that it is the neoepitope quality rather than its quantity which is, in fact, the major determinant of an effective immune response.

## 6. Blockade of Immune Checkpoints in Pancreatic Cancer

Immune-based therapy for pancreatic cancer has been taken into consideration in the last few decades, and consequently, the interest now created is fleeting. However, the response to these treatments is still inadequate, with 60–80% of patients not responding despite tumor regression and remission in some of the cases [128]. Moreover, since some tumors such as prostate and pancreatic cancer, which are quiescent types of tumors, are still resistant to these methods, new additional immunotherapy approaches are essential. [129]. The published clinical trials of checkpoint inhibitors are shown in Table 2.

## 7. Neoantigen Formation in Pancreatic Cancer

**Figure 2:**
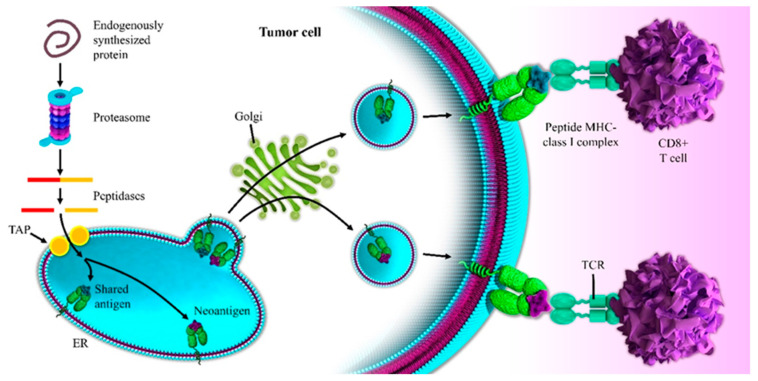
Neoantigen presentation to CD8+ T cells leads to higher TCR affinity, T-cell expansion, and higher numbers of TILs than the shared antigen presentation in pancreatic cancer (see text for the details).

The response to single-agent immune checkpoint therapies is reported for that 1% to 2% of patients with an unstable microsatellite phenotype. Therefore, what defines that resistance to therapy has become a huge area of investigation. It has mainly been thought that pancreatic cancer is a non-immunogenic tumor. This seems to be true if we look at the somatic mutation burden and the *x*-axis here, across the spectrum of different tumors, with classic UV- or tobacco-driven carcinogenesis in melanoma and lung cancer associated with hundreds, if not thousands of mutations. In actual fact, the historical data estimated only occasional neoantigen formation in pancreatic cancer [153]. However, pancreatic cancer is plagued by a typical extremely low tumor cellularity, making the sensitivity of DNA sequencing on bulk tumor material somewhat less than optimal. Recent sequencing efforts, including involving laser capture microdissected material or organoid expanded tumor epithelial material, suggested that pancreatic cancer may actually have a significantly higher rate of mutations, of over two mutations per megabase, which would convert pancreatic cancer up into a type of cancer which regularly and frequently generates an immune response [154]. Ultimately, pancreatic cancer might not be a completely mutationally silent tumor that does not potentially activate the immune system since plenty of mutations are present. Therefore, there are many alternative reasons why pancreatic cancer may be largely resistant to immune checkpoint therapy. Moreover, there are also features beyond simple neoepitope quantity as a driver of an operative immune response in PDAC [155]. An example is Balanchandran et al.’s comprehensive approach involving multiplexed immunohistochemistry for detailed immunophenotyping, TCR V beta sequencing to identify the clonal repertoire of T cells in each patient’s tumor, RNA-seq of bulk tumor material to characterize the intensity of the immune response, and whole-exome sequencing to recognize the burden of somatic mutations in a unique subset of long-term versus short-term pancreatic cancer survivors. The said researchers dissected the biological phenotype of all patients who had been surgically resected without prior chemo or radiation therapy, identifying a unique cohort of extraordinary pancreatic cancer survivors who had a minimum four-year survival after resection of their disease and with a significant number of 10-year survivors in this cohort [155]. In more detail, there were 80 patients in this cohort of extraordinary long-term pancreatic cancer survivors. The authors compared them to a staged matched cohort of patients who had a typical short-term survival. These were all stage and age and gender-matched. All had surgically resected primary tumors. None had metastatic disease at the time of resection, and no patients had neoadjuvant therapy [155]. Additionally, by multiplexed immunohistochemistry, Balanchandran et al. characterized the phenotype of any associated immune response, implementing the analysis with an immunofluorescent stain and strip and serial imaging and image registration technique. Overall, they found no difference in the absolute number of CD3 positive T cells between the short-term and the long-term survivors. Conversely, a slightly increased number of FoxP3 T-regulatory cells in the long-term survivors were observed, a slightly increased infiltration of dendritic cells, and an increased infiltration of myeloid cells. Of note, long term survivors displayed enhanced intratumoral T cell immunity. Specifically, looking at cytotoxic CD8 positive T cells, a threefold increase in CD8 positive T cells in the long- versus short-term survivors was observed; the authors uncovered a twelvefold increase in the number of cytolytic Granzyme-B positive, CD3 positive, CD8 positive T cells to be operative, suggesting that the long-term survivors display enhanced intratumoral T cell immunity. Wondering if the intratumoral T cells are tumor specific, TCR V beta sequencing mapped out the clonal repertoire of infiltrating T cells, not only in the tumor, but also in adjacent normal tissue in a subset of both short-term and long-term survivors. In both the short-term and long-term survivors, the T cell clones within the tumor were highly unique to the tumor. They were tumor-specific. Thus, in both groups, more than 95% of the T cell clones resided uniquely in the tumor rather than being shared with adjacent normal pancreas, suggesting that this infiltration might indeed be a specific T cell response to tumor-specific antigens rather than a nonspecific inflammation in the setting of adjacent pancreatitis. By looking at the clonality of the T cell repertoire in the long- versus short-term survivors, the investigators also found that the long-term survivors had a more diverse T cell repertoire than the short-term ones. Collectively, navigating the clonality, the long-term survivors had a more polyclonal, more diverse T cell infiltrate than the short-term survivors, and markers of overall immune activation using RNA-seq corroborated the enhanced intratumoral T cell immunity in the long-term survivors, which was displayed. The long-term survivors had a higher-level expression of PD1 up-regulated in the setting of an active immune response of TIGIT. They had up-regulated expression of dendritic cell markers and lower-level expression of immunosuppressive pathways, including STAT-3. Studies from our group could extend these findings by providing a deeper insight into the translational relevance of therapeutic dendritic cells and immune microenvironment targeting [156,157]. Our research was focused on tumors where more than 70% cellularity, either by microdissection or de novo, whole-exome sequencing and mutation identification, and then neoantigen prediction based on classic platforms that assess the binding of resulting peptides spanning somatic mutation to that patient’s HLA-typed MHC class I. In the frame of the above-mentioned results, the whole-exome sequencing identified putative neoantigens in PDAC, a very reasonable mutational load in these cancers in both the short- and the long-term survivors with an average of about two mutations per megabase, and about 70 non-synonymous mutations. While investigating which ones among these mutations were predicted to provide effective T cell neoepitopes based solely on MHC binding, a very reasonable burden of neoepitopes in this disease was found [155]. Finally, no difference in mutation or neoepitope burden between short- and long-term survivors was detected, with a combination of neoantigen burden and activated T-cells defining longest-term survivors. It is the neoantigen quality and not the quantity that stratifies long term survivors, being a prognostic impact in PDAC [155] as seen in Figure 2.

## 8. Conclusions and Future Perspectives

Unlike many cancers for which there is a dominant molecular subtype, pancreatic cancer tends to be a smear of mutation across a wide variety of genes in pathways, with the typical mutation frequency for any mutation in the 1% to 3% range [158]. While uncovering individual molecularly biomarker-driven therapy for pancreatic cancer, we have the difficulty of having no dominant clinical subset. We certainly do not have a dominant patient subgroup such as BRAF mutated melanoma or c-kit mutated GI stromal tumor, or EGF mutant lung cancer. In pancreatic cancer, it tends to be a smear. Therefore, this is going to continue to be a challenging area to apply molecular phenotyping to generate biomarker-driven therapy in this disease [159]. Long-term PDAC survivors represent a paradigmatic clinical phenotype to achieve a better deconvolution of the immune microenvironment and pancreatic cancer evolution, displaying evidence of enhanced T cell response. Enhanced T-cell response and prolonged survival are associated with unique neoepitope quality rather than quantity. Selection of patients for immunotherapy protocols would be of paramount importance, playing a pivotal role in the biomarker-driven selection of patients with gastrointestinal malignancies [160,161], for a whole variety of chemotherapy and targeted molecular and immune-based therapies. In the current study, we aimed to evaluate the outcomes of traditional chemotherapy in combination with targeted and immunotherapies. The result of the combinational therapies is still dismal in pancreatic cancer among the other malignancies; however, there is a higher survival rate among pancreatic cancer treated with targeted and immune based therapies in comparison to the chemotherapy alone. Among all these approaches, immune based therapies seem to be the most prospective approaches dealing with one of the most stubborn cancers. Hence, further investigation and research is needed in the field of newly merged therapies and evaluation of combinatorial effect of these therapies altogether to have a better prospect in pancreatic cancer treatment.

## Figures and Tables

**Table 1:** The role of immune checkpoint inhibitors and overall survival rates in different types of tumors.

Tumor Type	Immune Checkpoint Inhibitor	Overall Response Rate %
NSCLC	Nivolumab	20
RCC	Nivolumab	25
Melanoma	Nivolumab	32
Melanoma	Nivolumab + Ipilimumab	61
Melanoma	Pembrolizumab	33
Gastric/GEJ	Pembrolizumab	22
HCC	Nivolumab	20
MSI-H CRC	Nivolumab + Ipilimumab	81
MSS CRC	Pembrolizumab	0
MSI-H CRC	Pembrolizumab	40
Pancreatic	Nivolumab + Ipilimumab	1–2

Abbreviations: Non-small cell lung carcinoma (NSCLC), Renal cell carcinoma (RCC), Hepatocellular carcinoma (HCC), High microsatellite instability (MSI-H), Microsatellite instable (MSI).

**Table 2:** Published clinical trials of immune checkpoint inhibitors alone or in combination therapy.

Phase of Trial	Therapy	No of Patients	Setting	Median PFS/OS (Months)	Response	Reference
II	Ipilimumab	27	Locally advanced/metastatic 74% pretreated	OS: 4.5	0% RR 1 delayed response	[130]
I	BMS-936559	14	Advanced Pre-treated	NR	0% RR	[70]
II randomized	Durvalumab vs. Durvalumab + tremelimumab	33 32	Metastatic 2nd-line	OS: 3.6 OS: 3.1	6% DCR 9% DCR 3% PR	[131]
Pilot study	Durvalumab Durvalumab + tremelimumab + SBRT	24	Metastatic Pre-treated	NR	21% SD	[132]
II (safety profile)	Durvalumab + tremelimumab + gemcitabine + nab-paclitaxel	11	Metastatic No prior treatment	PFS:7.9	73% PR 100% DCR	[133]
Ib/II randomized	Pembrolizumab + capecitabine + RT vs. Capecitabine + RT	14 8	Neoadjuvant: 50% resectable/50% borderline resectable	NR	71% underwent surgery 50% underwent surgery	[134]
Ib dose escalation	Gemcitabine-> tremelimumab (different doses)	34	Metastatic No prior treatment	OS: 7.4	21% SD 6% PR	[135]
Ib	Ipilimumab + gemcitabine	16	Advanced No prior gemcitabine in advanced setting	PFS: 2.5 OS: 8.5	13% PR 31% SD	[136]
Ib/II	Pembrolizumab + gemcitabine + nab-paclitaxel	12 5	Metastatic No prior treatment Pre-treated	PFS: 9.1 OS: 15.0 NR	25% PR 67% SD 40% SD	[137]
II	Pembrolizumab + reolysin + 5-FU Pemrolizumab + reolysin + gemcitabine Pembrolizumab + reolysin + irinotecan	11	Metastatic 2nd line	NR	9% PR 18% SD	[138]
I	Nivolumab + nab-paclitaxel + gemcitabine	50	Locally advanced/metastatic No prior treatment PD-L1 expression ≥ 1%: 24% PD-L1 expression ≥: 12%	PFS: 5.5 OS: 9.9	2% CR 16% PR 46% SD	[139]
II, pilot	Nivolumab + nab-paclitaxel + gemcitabine + paricalcitol	10	Metastatic 1st line	PFS: 8.2	80% PR 100% DCR	[140]
Ib	Gemcitabine + nab-paclitaxel + APX005M (anti-CD40 antibody) ± nivolumab	30	Metastatic No prior treatment	NR	47% PR 27% SD	[141]
I Dose escalation	Gemcitabine Gemcitabine + 0.5 mg IMP321 Gemcitabine + 2.0 mg IMP321	6 6 5	Advanced 1st line	OS: 16.7 TTP: 10.2 OS: 5.6 TTP: 2.0 OS: 6.4 TTP: 5.3	83% SD 33% SD 60% SD	[142]
Ib randomized	Ipilimumab vs. Ipilimumab + vaccine	15 15	Advanced/metastatic Pre-treated	OS: 3.6 OS: 5.7	13% SD 0% SD	[143]
II randomized	Acalabrutinib vs. Pembrolizumab + acalabrutinib	26 32	Metastatic Pre-treated	NR	15% SD 9% PR 16% SD	[144]
Pilot study	Nivolumab + dendritic cells	7	Metastatic	NR	29% PR	[145]
I	Nivolumab + magamulizumab (anti-CC-chemokine receptor 4 antibody)	15	Advanced/metastatic	NR	7%PR 33% SD	[146]
I Dose escalation	Nivolumab + cabiralizumab (antibody directed against CSF-1 receptor)	31 evaluable	Advanced Pre-treated	NR	10% PR 3% SD	[147]
I/II	Oleclumab (antibody targeting CD73) ± durvalumab	20	Advanced Pre-treated	NR	10% PR 15% SD	[148]
I/II	Durvalumab + epacadostat	15	Advanced Pre-treated	NR	27% SD	[149]
Retrospective	pembrolizumab	2	dMMR	NR	50% PR 50% SD	[150]
II	pembrolizumab	8	Advanced Pre-treated dMMR/MSI positive	NR	25% CR 37% PR 12% SD	[151]
Retrospective	PD-L1 inhibitor + IDO1 (amino acid degrading enzyme) inhibitor or PD-1 inhibitor	7	Advanced Pre-treated dMMR	NR	14% CR 29% PR 14% SD	[152]

## Data Availability

Not applicable.

## Abbreviations

**Elaborated Phrasese**	**Abbreviations**
Pancreatic ductal adenocarcinoma	PDAC
programmed cell death protein-1	PD-1
programmed cell death ligand-1	PD-L1
cytotoxic T-lymphocyte-associated protein 4	CTLA-4
Guanine nucleotide exchange factors	GEFs
GTPase activating proteins	GAPs
mitogen-activated protein kinase	MAPK
extracellular signal-regulated kinase	Erk
phosphoinositide 3-kinases	PI3Ks
Phosphoinositide-dependent kinase-1	PDK-1
Ral guanine nucleotide exchange factors	RalGEFs
epidermal growth factor	EGF
epidermal growth factor receptor	EGFR
fibroblast growth factor	FGF
FGF receptor	FGFR
insulin-like growth factor	IGF
insulin-like growth factor receptor	IGFR
platelet-derived growth factor	PDGF
vascular endothelial growth factor	VEGF
transforming growth factor-α	TGF-α
Hypoxia-inducible factor 1-alpha	HIF1-α
Snail family of zinc finger transcription factors	Snail 1 and 2
zinc-finger-enhancer binding protein	Zeb-1 and 2
Casein kinase 1	CK1
lymphoid enhancer factor	LEF
tumor microenvironment	TME
antigen-presenting cells	APCs
interferon-γ	IFN-γ
Interleukin-2	IL-2
tumor-infiltrating lymphocytes	TILs
Lymphocyte Activation Gene 3	LAG3
basic helix-loop-helix	bHLH
Regulatory T cells	Tregs
plasmacytoid dendritic cells	pDCs
V-domain Ig suppressor of T cell activation	VISTA
Signal transducer and activator of transcription 3	STAT3
major histocompatibility complex	MHC
Nonsmall-cell lung carcinoma	NSCLC
Renal cell carcinoma	RCC
Hepatocellular carcinoma	HCC
High microsatellite instability	MSI-H
Microsatellite instable	MSI
carboxy terminal Cys2His2	C2H2
